# The prevalence of overweight/obesity and its association with household food insecurity among women living with HIV in rural Eswatini

**DOI:** 10.1186/s12889-022-13036-9

**Published:** 2022-03-31

**Authors:** Nozipho Becker, Andile Mkhonta, Lindiwe N. Sibeko

**Affiliations:** 1 Department of Nutrition, University of Massachusetts, Amherst, USA; 2 Department of Food and Nutrition Sciences, University of Eswatini, Luyengo, Kingdom of Eswatini; 3 Center for AIDS Prevention Studies, Division of Prevention Science, University of California, San Francisco, USA

**Keywords:** HIV/AIDS, Overweight, Obesity, Risk factors, Food insecurity, Nutrition-related noncommunicable diseases, NR-NCDs, Women, Eswatini, Sub-Saharan Africa

## Abstract

**Background:**

Eswatini is currently afflicted by an extremely high prevalence of HIV (27%) and malnutrition (both under-and over-nutrition). While rates of overweight/obesity in the general adult population have been documented, data on overweight/obesity and associated risk factors among women living with HIV (WLHIV) in Eswatini is limited. This study examines the prevalence of overweight/obesity and associated risk factors, with an emphasis on clarifying the association between household food insecurity and overweight/obesity for WLHIV in rural Eswatini.

**Methods:**

This cross-sectional study was conducted among WLHIV (*n* = 166) in rural communities of Eswatini. Data were collected using an interviewer-administered survey questionnaire between October and November, 2017. Body Mass Index (BMI) was calculated to determine overweight and obesity among study participants. Women with BMI values of 25 kg/m^2^ or greater were classified as being overweight/obese. Multivariable log-binomial regression models were used to examine associations between household food insecurity and overweight/obesity in our study.

**Results:**

Nearly a third (32.5%) of the women in our study were overweight and almost a quarter were obese (22.9%). We found significant associations between household food insecurity and overweight/obesity, with women who experienced household food insecurity the most being 0.38 times less likely to be overweight/obese compared to those who experienced household food insecurity the least (ARR: 0.38, 95% CI: 0.2–0.71). In our study sample, women who perceived themselves as being in poor health were less likely to be overweight/obese compared to those who perceived themselves as being in good health (ARR: 0.58, 95% CI: 0.39–0.86). We found significant associations between overweight/obesity and alcohol use, with the risk of overweight/obesity nearly 1.5 times higher among women who consumed alcohol compared to those who did not (ARR: 1.49, 95% CI: 1.07–2.05).

**Conclusions:**

The high prevalence of overweight/obesity among food insecure women in poverty stricken communities may pose significant challenges for nutritional health and HIV management. With an increasing prevalence of overweight/obesity in food insecure households, there is a need to re-evaluate current strategies and develop multi-level targeted interventions that include prevention of excessive weight gain among women, particularly those living with HIV in rural Eswatini. HIV programs could include screening to identify individuals at risk for overweight/obesity in this population, and provide nutrition education for weight management for those individuals.

## Introduction

In sub-Saharan Africa (SSA), rising prevalence of overweight/obesity co-exists with increased rates of nutrition-related noncommunicable diseases (NR-NCDs) [1]. Studies have shown that people living with human immunodeficiency virus (HIV) are just as susceptible to overweight/obesity as non-infected individuals, and are significantly impacted by the adverse effects of these health conditions [2]. Yet data on overweight/obesity and associated risk factors among people living with HIV in Eswatini are limited. As developing countries undergo shifts in dietary intake from traditional diets high in grains and fiber to western influenced diets high in fats and sugars, this nutrition transition has been associated with increased rates of overweight/obesity in these regions [1, 3, 4]. The rising prevalence of NR-NCDs as a result of the nutrition transition creates additional stress to already weakened immune systems of individuals living with HIV, thereby increasing the risk of morbidity and mortality among this population [2].

Eswatini is currently impacted by these health challenges. In addition to undergoing a nutrition transition, the country has been faced with a drastic increase in prevalence of overweight (28%) and obesity (23%), particularly among women [4]. In conjunction with high rates of overweight/obesity, Eswatini suffers from an extremely high prevalence of HIV (27%) [5] and malnutrition [6]. This increased risk of overweight/obesity may be further impacted by childhood experiences of undernutrition and recurrent infections, and collectively elevate the risk of NR-NCDs such as cardiovascular diseases, type 2 diabetes mellitus, and hypertension [2, 7, 8]. While the prevalence of overweight/obesity in the general adult population has been documented, no known studies have addressed overweight/obesity among women living with HIV (WLHIV) in Eswatini.

Food insecurity (FIS) is crucial to examine when addressing issues of nutritional status and health. Studies have shown that FIS is significantly associated with malnutrition (overnutrition/undernutrition) [9, 10], HIV acquisition and treatment outcomes [11, 12], and increased risk for NR-NCDs [2, 7]. FIS has been associated with increased consumption of calorie-dense foods, which are the main cause of overweight/obesity [1, 3]. Adverse dietary changes, including shifts in dietary patterns toward increased consumption of calorie-dense foods (e.g. foods high in saturated fats, added sugars and sodium) and reduced consumption of unprocessed nutrient-dense foods (e.g. whole grains, fruits and vegetables, and dietary fiber), combined with a reduction in physical activity, is associated with increased risk of NR-NCDs [1, 3]. While a consistent positive relationship between FIS and overweight/obesity has been reported among studies in developed countries overall [13, 14], reports from studies conducted specifically in SSA have been inconsistent, with some showing a positive association between overweight/obesity and FIS [15], and others reporting inverse associations [16–18].

A review of 19 studies by Franklin et al. (2012) examining the relationship between FIS and obesity suggest a strong positive association between FIS and excessive weight gain among women in the United States, even after adjusting for a wide range of socio-demographic characteristics [19]. This review is supported by several studies showing increased weight gain among food insecure individuals in developed countries. Among other factors, these studies suggest that this association may be due to increased poverty, sedentary lifestyle, and unhealthy food environments that facilitate access to calorie-dense foods [20–23]. Nkambule et al. (2021) reviewed 22 studies from countries in SSA. The purpose of the review was to assess associations between FIS and key metabolic risk factors among food-insecure individuals in the region. While findings from this review indicated positive associations between FIS and overweight, in the studies they reviewed, the authors observed a plethora of inconsistencies among previous studies, and noted the need for further research of the association between FIS and overweight in this region [24].

Socioeconomic status (SES) was frequently reported as a significant mediator of the observed associations between FIS and overweight/obesity. While some studies in SSA have reported positive correlations between overweight/obesity and high SES [25], others have reported similar associations among individuals of low SES [26]. Increased wealth and disposable income have a direct influence on food choices/dietary intake and are associated with increased consumption of calorie-dense foods, which in turn leads to increases in weight gain in the context of SSA [27]. Initially, in developing countries, this type of dietary shift and excessive weight gain was correlated with individuals of high SES [26]. However, recent findings from studies conducted in developing countries have been conflicting. Even though a majority of studies have found positive associations between high SES and overweight/obesity [26, 28], some have reported similar findings among individuals of low SES [29–31], and others found no significant associations between these groups, particularly among women [32, 33].

While the prevalence of overweight/obesity in the general adult population has been documented, no known studies have addressed overweight/obesity among WLHIV in Eswatini, who are currently the country’s most vulnerable to HIV infection and poor health [34–36].

Our study seeks to investigate the prevalence of overweight/obesity and associated risk factors, with a specific focus on clarifying the critical association between household food insecurity and overweight/obesity for WLHIV in rural communities of Eswatini. To our knowledge, this is the first study to investigate the prevalence of overweight/obesity and its association with food insecurity among WLHIV in rural Eswatini. This study provides scientific data that may help inform policies and intervention strategies aimed at preventing excessive weight gain and addressing issues of food insecurity among WLHIV, particularly those in resource limited settings.

## Methods

### Study design and setting

Data from this study were obtained from a cross-sectional study investigating barriers to antiretroviral therapy (ART) adherence among WLHIV in Eswatini [37]. The study was conducted in four rural communities located in the Lubombo and Shiselweni regions between October and November, 2017. This analysis examines the prevalence of overweight/obesity and associated risk factors, with an emphasis on clarifying the association between household food insecurity and overweight/obesity for WLHIV in rural Eswatini.

### Participants recruitment

Participants’ recruitment was conducted in collaboration with local health facilities and community health workers or expert clients. Study participants were recruited using targeted community techniques with both venue recruitment (attending community outreach activities and support group meetings) and invitation notices that were posted in public locations (e.g. health facilities, community centers, constituency buildings, churches, and neighborhood care points) within the respective communities. Women who were interested were invited to participate in a survey. Women were eligible to participate in the study if they were between ages 20–49 years, and had been on ART for at least one month at the time of the surveys. Participants were compensated with a 100 Rands for travel expenses and for time taken to participate in the study.

### Sample size

Based on previous literature, we estimated that approximately 60% of women living with HIV in our target communities would be overweight or obese. Using an alpha of 0.05 and a statistical power of 80%, 103 participants were determined to be the minimum sample required to effectively assess associations between food insecurity and overweight/obesity among women living with HIV in our study sample.

### Data collection

Data were collected through an interviewer-administered survey questionnaire. Study participants were interviewed in their native language of siSwati by trained research assistants. The surveys were conducted in a central location within respective rural communities, such as local constituency buildings, churches, and neighborhood care points. The survey questionnaire included the following items: 1) Household Food Insecurity Access Scale [38], 2) CASE Adherence Index [39], and 3) a structured questionnaire including demographic information and anthropometric measurements (age, weight, height). Weight was measured in kilograms (kg) using a digital bathroom scale, and height was measured in meters (m) using a stadiometer. Weight measurements were taken with participants wearing light clothing and barefoot.

### Outcome

Overweight and obesity were determined by using body mass index (BMI), calculated by dividing weight in kilograms by height in meters squared (kg/m^2^). BMI was categorized as underweight (< 18.5 kg/m^2^), normal weight (18.5–24.9 kg/m^2^), overweight (25–29.9 kg/m^2^), and obesity (≥ 30 kg/m^2^) [40]. The outcome was modeled as a dichotomous variable normal (18.5–24.9 kg/m^2^) versus overweight/obese (≥ 25 kg/m^2^).

### Exposure

Data on household food insecurity was collected using the Household Food Insecurity Access Scale(HFIAS) [38]. Participants were asked to provide information on their household food status in the month before the survey. Women were interviewed on all three domains of the HFIAS: 1) anxiety and uncertainty about the household food supply, 2) insufficient food intake, and 3) insufficient quality of food. Due to high prevalence of household food insecurity in the sampled communities, we observed an insignificant variation in the household food insecurity variable, as only 1.2% of the women were from food secure households. Therefore, food security data was used to assess two of the three HFIAS domains (i.e. insufficient food intake and insufficient quality of food) which were combined to create the food insufficiency variable. Women were classified as food insufficient if their answer to one or more of the food insufficiency questions was “yes”. Household food insufficiency was modeled as a dichotomous variable, women that were food sufficient versus women that were food insufficient. Given the high prevalence of food insecurity in our study population, we again observed an insignificant variation in the household food insufficiency variable, as only 8.4% of the women were found to be food insufficient.

Due to the extremely high rates of food insecurity observed in these communities, we decided to create a Household Food Insecurity Index variable, which is a comprehensive measurement adapted to capture the severity of food insecurity as it applies to our study population. The Household Food Insecurity Index consisted of three categories: 1) women who were from households that were food insufficient, 2) women who skipped taking medication due to hunger, and 3) those who missed doses due to hunger-related side effects from taking their medication. Next, composite scores were generated and calculated for each participant in our sample. Study participants received a composite score of 0, 1, 2, or 3 with 0 being the least food insecure and 3 the most. Cronbach’s alpha was used to determine the reliability of all items included in the household food insecurity index, and showed a reliability coefficient of 0.80, which is regarded as acceptable [41–44].

### Potential confounders

As part of the survey questionnaire, participants were also asked “In the past month, have you ever skipped taking your pills because of any of the following reasons: hunger, hunger-related side effects, poor treatment, forgetfulness, stress, clinic too far, no money for transport, etc.”. In the same questionnaire, participants were also asked to estimate travel time, transportation costs, and mode of transportation used to travel to and from the clinic or facility where they received health services. Travel time and transportation costs were modeled as continuous variables while mode of transport was modeled as a dichotomous variable, walking versus bus. Survey questionnaires were pilot tested prior to conducting this study.

### Data analysis

Data were analyzed using Stata version 17 (College Station, TX: StataCorp LP). Data analyses were conducted at the descriptive, bivariate, and multivariable levels. Continuous variables are presented as means with standard deviations, and categorical variables are presented as frequencies and percentages. The majority of categorical variables were normally distributed. For continuous variables, the Kolmogorov-Smornov and Shapiro–Wilk tests were conducted to determine whether data was normally distributed. Travel time was not normally distributed, therefore data were transformed to its natural logarithm. Differences in socio-demographic characteristics between women who were overweight/obese versus those who were normal weight were assessed using Pearson’s chi-squared test of significance for categorical variables, and the t-test for continuous variables. Variables that were significantly associated with overweight/obesity at a significance level of 25% were selected and included in multivariable regression models.

The variable “Household Food Insecurity Index” was used as the main exposure variable, having a wider distribution and demonstrating a better fit for the model. Multivariable log-binomial regression models were used to assess the relationship between overweight/obesity and household food insecurity, while controlling for potential confounders in the model. Variables were considered statistically significant when ***p*** < 0.05.

## Results

The study population consists of 166 women living with HIV in rural communities of Eswatini (Table 1). Of the 166 participants, 44.6% were normal weight, 32.5% were overweight, and 22.9% were obese. A majority of the participants were married/living with a partner (56.6%), with participants mean age of 37 years. Of the 166 participants, 23.5% had no formal education, while 76.5% attended some primary and/or high school education. A striking 54.8% of the participants were unemployed, with 45.2% of the participants reporting being employed full-time or limited to seasonal or part-time employment at local clothing factories and sugarcane fields. Of the 166 women, 51.2% occasionally obtained food and/or financial support from family members (including husbands, partners, and relatives), neighbors, friends, and community members, while 48.8% reported not receiving any form of support.

**Table 1:** Demographic characteristics of study participants, *n* = 166^a^

Characteristic	N	%	Mean (SD)
Age	166		37.1 (7.3)
Travel time	149		1.9 h (1.7)
**Body mass index (kg/m**^**2**^**)**			
Normal (18.5–24.9)	74	44.6	
Overweight (25–29.9)	54	32.5	
Obese (> 30)	38	22.9	
**Educational level**			
No school	39	23.5	
Primary/high school	127	76.5	
**Marital status**			
Not married	72	43.4	
Married/ living with partner	94	56.6	
**Employment status**			
Not employed	91	54.8	
Employed	75	45.2	
**Household income (Rands)**			
< = 500	91	55.1	
500 +	74	44.9	
**Food insecurity index**			
0 (least food insecure)	7	4.2	
1	23	13.9	
2	39	23.6	
3 (most food insecure)	96	58.2	
**Alcohol use**			
None drinkers	130	78.8	
Drinkers	35	21.2	
**ART adherence status**			
Adherent	73	44.0	
Nonadherent	93	56.0	
**Health status**			
Good	116	69.9	
Poor	50	30.1	
**Transport mode**			
Walking	110	66.3	
Vehicle	56	33.7	
**Family support**			
No	80	48.8	
Yes	84	51.2	

^a^Sample sizes for variables may not add up the total due to missing data

Table 2 presents measures of associations between overweight/obesity and socio-demographic factors. Overweight/obesity was significantly higher among women who reported being in good health compared to those who reported being in poor health (64.7%, *p* = 0.000). We found significant associations between overweight/obesity and household food insecurity, with prevalence rates being significantly higher among women who experienced food insecurity the least compared to those who experienced it the most (85.7% versus 44.8%, *p* = 0.003). Similarly, the prevalence of overweight/obesity was higher among women who traveled for shorter periods of time to and from the clinic compared to those who traveled for longer periods of time (54.4%, *p* = 0.04).

**Table 2:** Measures of association between overweight/obesity and associated risk factors

Risk factors	Normal weight		Overweight/obesity		*P*—value
	**N**	**%**	**N**	**%**	
Age	75	45.1	91	54.8	0.976
Travel time	68	45.6	81	54.4	0.041
**Educational level**					0.612
No school	19	48.7	20	51.3	
Primary/high school	56	44.1	71	55.9	
**Marital status**					0.882
Married/ living with partner	42	44.7	52	55.3	
Not married	33	45.8	39	54.2	
**Employment status**					0.366
Not employed	44	48.4	47	51.6	
Employed	31	41.3	44	58.7	
**Household income (Rands)**					0.316
< = R500	44	48.4	47	51.6	
R500 +	30	40.5	44	59.5	
**Food insecurity index**					0.003
0 (least food insecure)	1	14.3	6	85.7	
1	4	17.4	19	82.6	
2	16	41.0	23	59.0	
3 (most food insecure)	53	55.2	43	44.8	
**Alcohol use**					0.135
Nondrinkers	63	48.5	67	51.5	
Drinkers	12	34.3	23	65.7	
**ART adherence status**					0.349
Adherent	30	41.1	43	58.9	
Nonadherent	45	48.4	48	61.6	
**Health status**					0.000
Good	41	35.3	75	64.7	
Poor	34	68.0	16	32.0	
**Transport mode**					0.448
Walking	52	47.3	58	52.7	
Vehicle	23	41.1	33	54.8	
**Family support**					0.078
No	30	37.5	50	62.5	
Yes	43	51.2	41	48.8	

Similarly, in multivariable adjusted regression models (Table 3), we found significant associations between household food insecurity and overweight/obesity. Women who experienced household food insecurity the most were 0.38 times less likely to be overweight/obese compared to those who experienced household food insecurity the least (ARR: 0.38, 95% CI: 0.2–0.71). In our study population, the risk of overweight/obesity was significantly associated with perceptions of health status. Women who perceived themselves as being in poor health were 42% less likely to be overweight/obese compared to those who perceived themselves as being in good health (ARR: 0.58, 95% CI: 0.39–0.86). We found significant associations between overweight/obesity and alcohol use, with the risk of overweight/obesity nearly 1.5 times higher among women who consumed alcohol compared to those who did not (ARR: 1.49, 95% CI: 1.07–2.05). In addition, women who felt supported by family members were 27% less likely to be overweight/obese as those who did not feel supported by their families (ARR: 0.73, 95% CI: 0.53–0.99).

**Table 3:** Results of multivariable analysis of the association between overweight/obesity and household food insecurity among women living with HIV in rural Eswatini

Risk Factors	*z*-value	Adjusted Risk Ratio (ARR)	95% CI
Age (years)	1.87	1.02	(0.99–1.04)
Food insecurity index			
0 (least food insecure)	reference	1.00	(-)
1	-0.62	0.82	(0.45–1.52)
2	-1.66	0.62	(0.35–1.09)
3 (most food insecure)	-3.06*	0.38	(0.21–0.71)
Alcohol use (no vs. yes)	2.41*	1.49	(1.07–2.05)
Health status (good vs. poor)	-2.74*	0.58	(0.39–0.86)
Gossip (no vs. yes)	3.36	0.91	(0.73–1.13)
Family support (no vs. yes)	-2.01*	0.73	(0.53–0.99)

*z*-value: test statistic; *CI* confidence interval; ^*^p < 0.05

## Discussion

This study examined the prevalence of overweight/obesity and its association with household food insecurity among WLHIV in rural Eswatini. We found significantly higher rates of overweight/obesity among women reporting the lowest levels of food insecurity in our study, suggesting an inverse association between food insecurity and weight gain. Other studies in the region have reported similar findings, where lower rates of overweight/obesity were found among food insecure individuals [17, 18]. In a study conducted among women in Kenya, food insecurity was found to be a significant predictor of overweight/obesity, with higher rates observed among women in urban areas as opposed to those in rural areas [18].

Among studies in sub-Saharan Africa, household food insecurity has been adversely associated with an increased prevalence of multiple metabolic risk factors including overweight, obesity, dyslipidemia, and hypertension [24]. The impact of food insecurity on food choices, dietary intake, and nutritional status has long been recognized [21, 45, 46]. Potential mechanisms explaining the association between food insecurity and increased rates of overweight/obesity in our study may include the following: 1) an increase in consumption of diets comprised of high calorie foods in rural households (e.g. processed grains, fats/oil, and sugars) [1, 24, 27], 2) chronic periods of inadequate dietary intake/hunger combined with compensatory overconsumption of high calorie foods, potentially triggering the thrifty phenotype phenomenon among our study population [47], and 3) changes in dietary patterns as a result of nutrition transition with increased access to and availability of processed/calorie dense foods in these communities [1, 48].

Nearly a third of the women in our study were overweight and almost a quarter were obese. While the prevalence of overweight is comparable to those reported in previous studies, the rates of obesity in our study were slightly higher than those reported among women in the general population of Eswatini [4]. The high rates of overweight/obesity observed among women living with HIV in our study are alarming, particularly due to their association with increased risk of NR-NCDs [2, 9, 10, 24]. Furthermore, our findings show significantly higher rates of overweight/obesity among women who perceived themselves as being in good health. In many SSA cultures, weight gain is traditionally associated with perceptions of wealth and good health. Overweight/obesity is culturally accepted, often deemed desirable, and typically perceived as ‘normal’ (especially among women) [49–51].

In some African countries where HIV/AIDS is highly prevalent, a larger body size is viewed as a sign of good health and thereby associated with being free of HIV infection. In a study conducted among women in South Africa, being overweight/obese was preferred by a more than a third of the women and regarded as indicative of health, while a thinner frame was often associated with a higher likelihood of that person having HIV/AIDS [52]. In another study conducted among female community health workers in South Africa, being overweight was preferred and often associated with dignity, respect, confidence, beauty, and wealth [51]. Similarly in Eswatini, being overweight is often perceived as a sign of wealth and life success [53], though it is unclear whether people are actively gaining weight as a way to achieve societal acceptance and appear healthy (particularly in regard to HIV status). Studies have shown that culture/traditional beliefs are crucial to examine when addressing issues of health and practices related to food and eating patterns, as well as attitudes and perceptions about body weight and disease [49, 51]. Given that cultural body size preferences have been shown to influence nutritional health and disease outcomes, there is a need to investigate this association further in the context of the Eswatini.

We found significant associations between alcohol use and an increased risk of overweight/obesity. Alcohol use has been documented as a widely used means of coping with a range of stressors including economic stress, marital problems, and lack of social support [54–56]. Empirical evidence indicates that the more severe and chronic the stressor, the greater the alcohol consumption [54–56]. In Eswatini, 59% of those who drink regularly consume a homemade traditional brew, commonly known as *umcombotsi* [57]. *Umcombotsi* is an inexpensive (approximately $0.25 USD per liter) alcoholic beverage typically served at local drinking spots (*shebeens*) throughout rural communities of Eswatini. In our previous study of barriers to ART adherence, women reported excess alcohol use as a common strategy for coping with stress and hunger [12]. The current study findings are parallel to other studies that have documented associations between alcohol consumption and overweight/obesity among countries in SSA [58]. High levels of food insecurity and stress combined with easily available alcohol create enormous problems in communities already burdened by poverty and chronic diseases.

To our knowledge, this is the first study to investigate the prevalence of overweight/obesity and its association with household food insecurity among WLHIV in Eswatini. In addition, our study generated important data regarding variations in prevalence by socio-demographic factors, thereby providing us with a suggestive evidence of potential risk factors of overweight/obesity in this population. Our study may be limited due to minimal sample size, which may have led to an underestimation of the reported prevalence rates and degree of association between household food insecurity and overweight/obesity. In addition, this study relies on self-reported data which may be susceptible to human error and may be influenced by recall bias. In order to minimize this potential limitation, we used an objective measurement of BMI that was computed from weight and height of participants that were directly measured rather than reported. Despite the limitations, this study provides important information on the prevalence of overweight/obesity and associated risk factors among WLHIV in Eswatini, who are currently one of Eswatini’s most vulnerable sub-populations to infections and poor health outcomes. In addition, our study generated important data regarding variations in overweight/obesity prevalence by food insecurity status, thereby providing us with a glimpse of potential risk factors of overweight/obesity in this population.

## Conclusions

The prevalence of overweight/obesity among women in poverty stricken communities may pose significant challenges for nutritional health and HIV management. The direct impact of food insecurity on dietary intake and poor health outcomes has long been recognized. Food insecurity not only impacts the ability to consume adequate nutrients, it also increases a person’s risk of poor nutritional health and susceptibility to infections including HIV. In Eswatini, there has been a drastic increase in overweight and obesity rates over the past years, with women more overweight/obese than men [59]. Overweight and obesity have been found to be significant predictors of numerous NR-NCDs including type 2 diabetes mellitus, hypertension, cardiovascular disease, and certain cancers [1, 60, 61]. The increasing rates of overweight/obesity among WLHIV in rural households are disconcerting as they suggest an increased risk of NR-NCDs and further exacerbating challenges experienced by women in these communities [60, 61]. With an increasing prevalence of overweight and obesity in food insecure households, there is a need to re-evaluate current strategies and develop multi-level targeted interventions that include prevention of excessive weight gain among women, particularly those living with HIV.

## Data Availability

The datasets generated and/or analyzed during the current study are available in the Figshare repository, https://doi.org/10.6084/m9.figshare.12174273

## Abbreviations

SSA: Sub-Saharan Africa
NR-NCDs: Nutrition-Related Noncommunicable Diseases
HIV: Human Immunodeficiency Virus
WLHIV: Women Living with HIV
FIS: Food Insecurity
ART: Antiretroviral Therapy
BMI: Body Mass Index
Kg: Kilograms
M: Meters
HFIAS: Household Food Insecurity Access Scale
ARR: Adjusted Risk Ratios
CI: Confidence Interval
